# Effects of augmented feedback delay on neural correlates of feedback processing in motor practice

**DOI:** 10.1007/s00221-026-07365-z

**Published:** 2026-08-24

**Authors:** Linda Margraf, Daniel Krause, Lisa Katharina Maurer, Matthias Weigelt

**Affiliations:** 1 Psychology and Movement Science, Department of Sports and Health, Paderborn University, Warburger Str. 100, Paderborn, Germany; 2 Training and Movement Science, Physical Education and Sports Science, University of Kassel, Damaschkestr. 25, Kassel, Germany

**Keywords:** Event-related brain potentials, Delayed augmented feedback, Feedback valence, Error processing, Motor practice

## Abstract

Distinct neural correlates are associated with the processing of augmented feedback during motor learning. To examine the basic neural response of delayed augmented feedback, 33 participants practiced a right-arm flexion-extension-movement performed with an arm-lever-device in 192 trials with subsequent feedback presentation. The movement sequence consisted of three movement reversals, which were practiced with the goal of minimizing errors in spatial accuracy. Based on a performance-adaptive bandwidth for spatial movement accuracy, participants received delayed augmented feedback indicating whether they had achieved one predefined reversal (of the three), or not. The feedback delay duration alternated block-wise between 1500 ms and 7000 ms. EEG was recorded throughout practice. The error-related negativity (ERN) and the error positivity (Pe) are assumed to reflect neural error processing in a predictive manner based on intrinsic information, whereas the feedback-related negativity (FRN) is assumed to reflect neural error processing in a postdictive manner based on extrinsic outcome feedback. It was hypothesized that the ERN and the Pe would be more pronounced in trials with longer feedback delays, which unfortunately could not be tested. The relevant components were not detected in the signal, possibly due to the complex sequential structure of the task and the feedback pattern. With respect to feedback valence, results regarding the FRN were as expected. Further, the results demonstrate a more pronounced neural processing of augmented feedback with respect to a shorter feedback delay duration. These results indicate that delaying augmented feedback impacts the neural processing of augmented feedback in motor practice.

**Supplementary Information:**

The online version contains supplementary material available at https://doi.org/10.1007/s00221-026-07365-z.

## Introduction

Humans use feedback to evaluate their actions and their outcomes to optimize behavior. In doing so, they rely either on the body’s own intrinsic information or on perceptible effects in the environment that are triggered by the action. In the context of motor practice, additional augmented feedback (defined as additional information that is not available in the natural task setting, supplementing natural sensory intrinsic feedback; e.g., extrinsic feedback information about the performance provided by a coach) is often implemented to facilitate learning (e.g., Magill and Anderson 2014; Weigelt et al. 2023). However, when designing feedback-based practice sessions, there are various factors that can be manipulated, among these is the timing of augmented feedback information. For example, a coach can provide augmented feedback about success or failure (error feedback) of a given motor task immediately after its completion or with a shorter or longer delay between action completion and feedback presentation (i.e., feedback delay). In the motor domain, short feedback delays are defined as those lasting up to five seconds, while delays exceeding this duration are classified as long (Travlos and Pratt 1995). Although individual studies have applied either short (e.g., 3.2 s) or long (e.g., 8 s) feedback delays (Swinnen et al. 1990) and compared it to immediate feedback (i.e., no delay of feedback), there is no data on a systematic comparison of shorter and longer feedback delays within a single study. Numerous studies have examined the effects of feedback delay duration on the acquisition of a motor task based on behavioral measures (for a meta-analysis, see Travlos and Pratt 1995), while the impact on underlying cognitive mechanisms of feedback- and error-processing is not yet clarified. Therefore, it appears reasonable to further investigate feedback delay effects in motor practice by supplementing the behavioral findings with an examination of neural indicators related to feedback-based motor practice. The present study, therefore, focuses on the basic neural response to different feedback delay durations based on selected event-related potentials (ERPs) of the human electroencephalogram (EEG), which have been substantially examined in the cognitive domain (Hinneberg and Hegele 2022), but not in the motor domain.

From a behavioral perspective, studies on the timing of augmented feedback have shown that immediate versus delayed feedback often produces comparable short-term performance changes within single practice sessions (Schmidt and Lee 2011). In contrast, analyzing long-term learning, research in the cognitive domain (e.g., Brackbill et al. 1964; Mullaney et al. 2014; Schroth 1992) and occasionally in the motor domain (e.g., Swinnen et al. 1990; Travlos and Pratt 1995) suggests that short feedback delays may even facilitate retention performance as compared to immediate feedback (delay-of-feedback benefit), especially when augmented feedback is withdrawn in retention tests.

According to the Guidance Hypothesis (Salmoni et al. 1984), immediate feedback hinders the development of error-detection capabilities because it reduces the learner’s reliance on intrinsic feedback. Instead, learners tend to depend excessively on augmented feedback as their primary source for movement correction (Liu and Wrisberg 1997). This over-reliance occurs because immediate feedback interferes with the processing of intrinsic sensory information; specifically, intrinsic cues require time to be processed and integrated into declarative memory, a process that is disrupted when immediate external feedback is provided. However, during the feedback delay interval, the sensory consequences must be retained in memory until the augmented feedback information is available, so that both can be compared (Schmidt and Lee 2011). It is important to develop own error-detection capabilities based on inherent feedback sources. To build up error-detection capabilities, it seems advantageous if there is a subjective error estimation during the feedback delay interval during acquisition (Swinnen 1990; Swinnen et al. 1990). At this point, the issue of the duration of the feedback delay and its impact on information processing arises. According to reinforcement learning models (Holroyd and Coles 2002), feedback processing involves dynamic integration of internally generated prediction (based on efference copies and proprioceptive feedback) and externally provided augmented feedback information. The temporal alignment of these signals is crucial for effective credit assignment and error correction. Feedback delay potentially impairs this integration process by disrupting the temporal contiguity between intrinsic predictions and augmented feedback, leading to modulation of neural markers. The incorporation of such models clarifies the differential impacts of feedback delay on implicit (unconscious) versus explicit (attention-dependent) processes. Thus, short feedback delay durations should favor implicit reinforcement learning processes, while longer feedback delay durations should promote the development of one’s own error-detection capabilities and explicit error processing based on intrinsic feedback information.

Neurophysiologically, distinct ERP components are associated with different neural mechanisms of feedback processing and may thus be modulated by the feedback delay duration (e.g., Höltje and Mecklinger 2020; Opitz et al. 2011; Paul et al. 2020; Weinberg et al. 2012). The feedback-related negativity (FRN), peaking approximately 200–300 ms after feedback onset at frontal sites, shows more negative activation after negative feedback (for a meta-analysis in the cognitive domain: Sambrook and Goslin 2015; for a meta-analysis in the motor domain: Faßbender et al. 2023). In this context, it is important to note that the terminology used for this component is not entirely consistent. In place of the designation FRN, the component has been argued to be appropriately referred to as reward positivity (RewP). This is due to the premise that rewarding feedback would serve to mitigate the negative response to feedback, as indicated by the N200 (for more detailed information about this rationale: Krigolson 2018; Proudfit 2015). In the context of the present study, however, the term FRN is used because it is more widely used in the motor domain (Faßbender et al. 2023). The FRN is quantified as the difference wave (negative minus positive feedback) and is assumed to reflect rapid implicit evaluation of outcome valence and prediction errors in reinforcement learning, mediated by phasic activity of midbrain dopamine neurons and respective projections to the striatum and the dorsal anterior cingulate cortex (e.g., Gehring & Willoughby 2002). Although the dorsal anterior cingulate cortex is assigned to the attentional control network (Chein and Schneider 2012), the component is associated to implicit reinforcement learning processes (Höltje and Mecklinger 2020). This discrepancy might be resolved by assuming two overlapping frontal components in the respective time-window (Höltje and Mecklinger 2020; Peterburs et al. 2016): The FRN_diff_ (quantified as the peak in the negative minus positive difference wave) is considered to reflect implicit reinforcement learning, while the FRN_peak_ (peak-to-peak difference of the N200 minus the preceding P200 in the valence-specific waveforms) is associated to attention-dependent processes, especially after negative prediction errors. In the following, the term FRN refers to the quantification as FRN_diff_. Critically, the FRN is sensitive to temporal alignment of predicted and received feedback, hence, FRN amplitudes are modulated by feedback delay (for a review, see Hinneberg and Hegele 2022): Short delays (i.e., 500–1000 ms) elicit larger amplitudes than longer delays (i.e., 6500–7000 ms) in cognitive tasks such as artificial grammar learning, probabilistic learning, or time estimation (Arbel et al. 2017; Höltje and Mecklinger 2020; Opitz et al. 2011; Paul et al. 2020; Peterburs et al. 2016; Weinberg et al. 2012; Weismüller and Bellebaum 2016). However, this effect has not yet been examined in the motor domain. Moreover, Weismüller & Bellebaum (2016) present data suggesting that the neural mechanism eliciting the FRN component are identical for immediate and delayed feedback. Further, a study from Kimura et al. (2016) suggests that temporal prediction had a high impact on neural processing. Unpredictability of the feedback delay appears to result in smaller FRN amplitudes.

All studies cited here use decision making or time-estimation tasks, that are not representative for motor-learning-task settings with a substantially more complex structure of sensory information as a basis for predictive and postdictive processing of integrated intrinsic and augmented feedback (Maurer et al. 2022). A substantially more ambiguous mapping of augmented feedback to intrinsic sensory feedback and the respective relevant motor elements of the task is inherent to motor-learning-task settings (Krause et al. 2020; Margraf et al. 2022 ) and clearly distinct from tasks with a minimal involvement in the cognitive domain (e.g., time estimation, gambling and decision making). Moreover, domain specific characteristics of feedback processing have been reported on a meta-analytic basis (Fassbender et al. 2023). Therefore, it seems reasonable to test generalizability of findings of the effects for feedback delay on feedback processing in the motor domain.

Action outcomes can also be detected and evaluated prior to external feedback, using internal forward models that integrate efference copies of motor commands with intrinsic feedback (e.g., proprioceptive feedback) to predict the outcome of the current movement with respect to success or failure (Miall and Wolpert 1996; Shadmehr et al. 2010). Two ERP-components are relevant here: the error-related negativity (ERN) and the error positivity (Pe). The ERN is a fronto-central negativity emerging about 100 ms after a motor action is executed, reflecting early error detection (Falkenstein et al. 1991). The ERN has been observed across motor tasks varying in complexity, including tracking (Krigolson and Holroyd 2006), goal-directed throwing (Maurer et al. 2015, 2022), and sequence learning (Beaulieu et al. 2014). The Pe is a later component with a slow positive wave occurring between 200 and 600 ms above centro-parietal electrodes and is linked to error awareness (Endrass et al. 2012; Falkenstein et al. 1991; Overbeek et al. 2005; Ridderinkhof et al. 2009). The evidence with respect to the impact of feedback delay duration on the ERN and Pe is still unclear. However, it is theorized that longer feedback delays shift reliance towards explicit error processing which may be reflected in enhanced ERN and Pe amplitudes.

The aim of the current study is to investigate how feedback delay duration affects the neural processing of valence-dependent augmented feedback (FRN), and in an explorative manner, the neural error evaluation based on intrinsic feedback information prior to outcome feedback (ERN, Pe). Basic neural findings (cf. Hinneberg and Hegele 2022) should be replicated for the first time in the motor domain. In this context, this study also serves as a preliminary investigation with the objective of examining the basic neural response to varying durations (short, long) of feedback delay.

The current study contained one experimental session in which participants practiced a sequential motor task. The task was a right-arm extension-flexion-movement comprising three movement reversals performed with a lever device (e.g., Krause et al. 2020; Margraf et al. 2022). Feedback was given after every practice trial based on a performance-adaptive bandwidth (a predefined range of tolerance around the target value within which positive feedback is provided, e.g., Agethen & Krause, 2016; Weigelt et al. 2020). In each trial, feedback was given for one of the three reversals. The delay after which the feedback was given after reaching the relevant movement reversal was changed block-wise (short feedback delay: 1500 ms, long feedback delay: 7000 ms).

With respect to the motor performance, it is expected that participants will improve their performance during the practice in the experimental session. If this were the case, their performance should be more accurate (absolute error, *H.AE*) and more consistent (variable error, *H.VE*) in the post-test at the end of the experimental session, as compared to the pre-test before practice. Further, it is hypothesized that short feedback delays result in activation processes of reinforcement learning and, therefore, elicit higher amplitudes of the FRN (*H.FRN)* as compared to long feedback delays. In contrast, longer feedback delays are associated with better preconditions for error detection. On an explorative basis, it could be assumed that longer feedback delays induce a higher ERN (*H.ERN*) and a higher Pe (*H.Pe*) as compared to short feedback delays.

## Methods

### Participants

Forty participants were tested (23 females; mean age = 21.18 ± 2.41; age range = 19–31 years). Four participants were excluded from later analysis due to excessive EEG artifacts and three participants due to being behavioral outliers (> 3 SD from the mean). The final analyses comprised 33 undergraduate students (17 females; mean age = 21.36 ± 2.55; age range = 19–31 years). Of these participants, 28 were right-handed, as assessed with the German version of the Edinburgh Handedness Inventory (Büsch et al. 2010). All had normal or corrected-to-normal vision, no upper-limb lesions, provided written informed consent, and received course credits.

### Apparatus and task

**Fig. 1:**
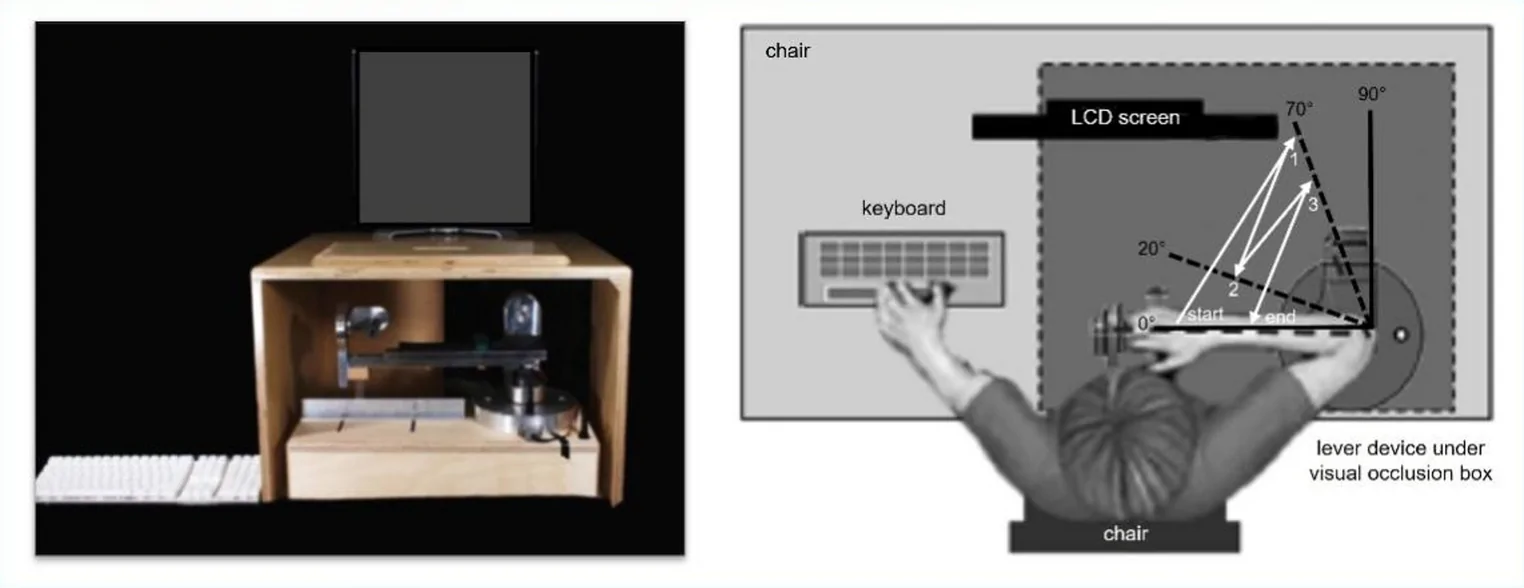
Apparatus and experimental setup. The apparatus can be found on the left side. The arm-lever device is placed under a wooden box which prevents visual movement control and serves also as a podium for a monitor. A keyboard serves as input device for the participant. The experimental setup is shown on the right side. A participant is sitting in front of the setup on an adjustable chair; the right arm is placed on the lever-device and the other is placed on the keyboard. The target angles are marked, and the order in which they should be hit is indicated schematically by the white lines. (reprinted from Margraf et al. 2022, p. 9. Copyright 2022, with permission from Elsevier).

The experimental setup is shown in Fig. 1. Participants performed a right-arm elbow extension-flexion sequence with three reversals (70°, 20°, 70°, starting at 0°-position) using an adjustable underarm lever device enabling horizontal rotation. Elbow angles were measured via a linear potentiometer (P6501, Novotech 6500) powered at 6 V (Voltkraft PS 1152 A) and digitized (NI USB-6001). Data were acquired with Dasylab 2022 and converted to angular degrees for reversal detection and movement time calculation. Custom-build Java software (PaDuTas) controlled movement sequences and feedback presentation. Participants aimed to hit the reversals as precisely as possible while keeping movement time under 1200 ms. The sequential movement was finished by crossing the 0°-position without stopping.

### EEG recordings

 EEG was recorded using a 64-channel AC/DC amplifier with electrodes based on Ag/AgCl sensors (eego EE-225, ANT). The scalp electrodes were applied with an electrode cap (waveguard original cap, ANT Neuro) according to the international 10–20 system. Impedances of the electrodes were kept below 10kΩ. The ground electrode was set on AFz, online reference on CPz. An EOG electrode below the left eye monitored vertical eye movements. EEG data was collected on a separate laptop at a sampling rate of 500 Hz (eego software, ANT Neuro).

The EEG was synchronized to feedback onset via a photodiode (XS-355, ANT Neuro ), attached to a 1.5 cm^2^ area in the right lower corner of the feedback screen which marked the onset of feedback presentation. The area was shielded with adhesive tape. Brightness of this screen area changed from dark to light with feedback presentation. Data of the photodiode was sampled via sense box (XS-271.A, ANT Neuro) to the amplifier.

**Fig. 2:**
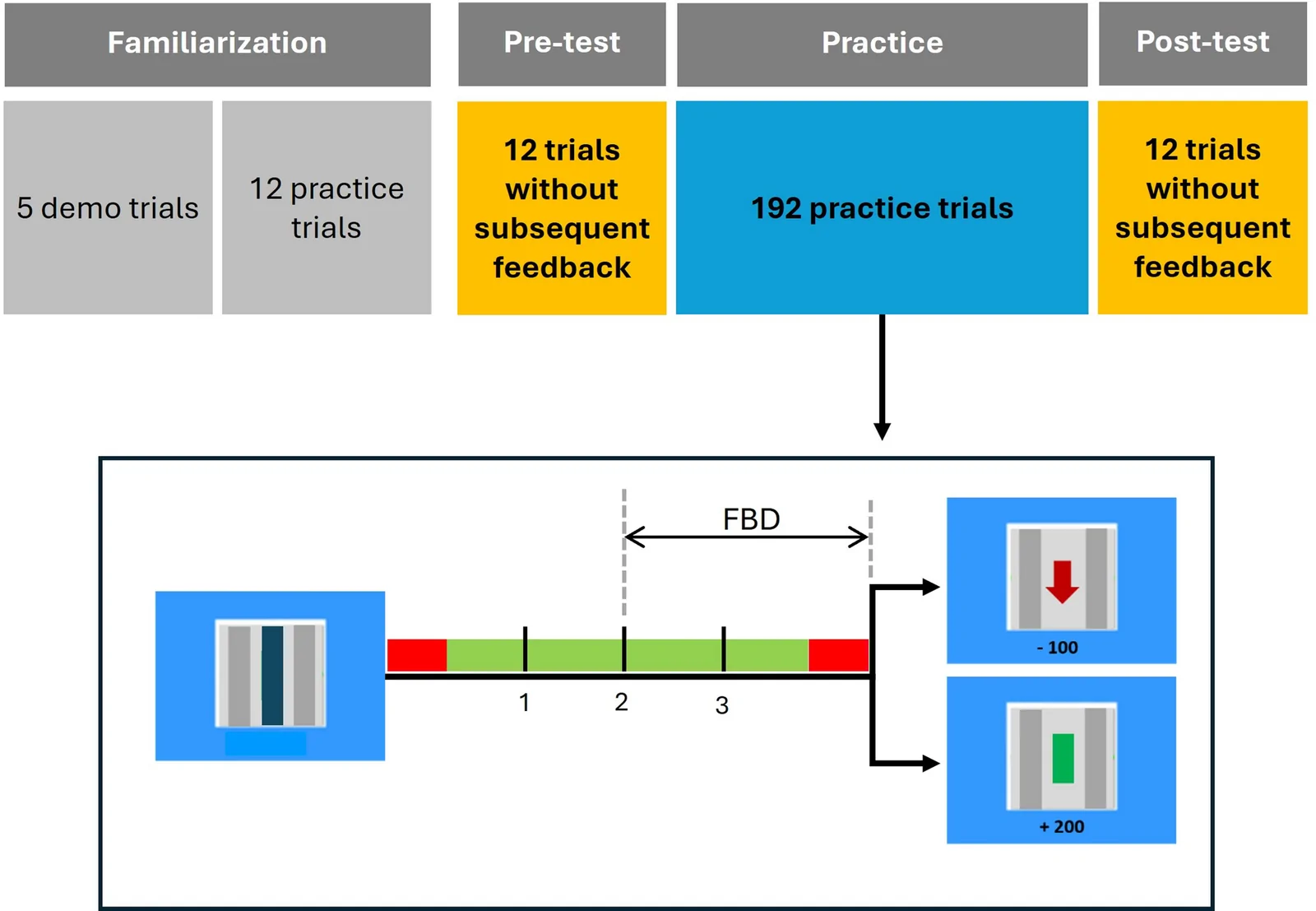
Overview of the experimental setup. The session started with a familiarization phase consisting of 5 demo trials with subsequent feedback for each of the three reversals, followed by 12 practice trials in which the feedback was reduced to one predefined reversal indicated by a blue bar as displayed in the box on the left side. The addressed reversal changed block wise. Afterwards, 12 trials without subsequent feedback were conducted as a pre-test. The following practice phase consisted of 8 blocks of 24 trials each. After a short or long delay (changing also block wise), augmented feedback was presented after every trial for the addressed reversal. The counter for the feedback delay (FBD) started when the defined reversal was reached. The feedback was based on a performance-adaptive bandwidth calculated from the median of the last 12 trials. A performance within the actual bandwidth was rewarded with positive feedback and 200 points, displayed in the box on the right side in the bottom row. A performance outside of the bandwidth led to negative feedback and a loss of 100 points, displayed in the box on the right side in the upper row. The direction of the arrow indicates the direction of the error. If the angle was too small, the arrow points downwards, if the angle was too large, the arrow points upwards. The session ended with another 12 trials without subsequent feedback as a posttest

### Procedures

 The experiment consisted of one experimental session of approx. 2.5 h; an overview can be found in Fig. 2. The session began with informed consent, the General Data Protection Regulation, and a questionnaire (demographics, vision, limb injuries, handedness). The instruction of the task was done via presentation slides (Power Point, Microsoft) and standardized verbal guidance.

The criterion motor task was explained and demonstrated to the participant by an avatar demonstrating the movement five times (demo trials). After each demonstration the participants were asked to execute the movement themselves (the instruction slides including the video of the avatar performing the motor sequence can be found here: https://osf.io/swj4c). Trials started with a start-position cue (1500 ms), followed by a 3000-ms countdown (“3-2-1”) after which a bar at the upper edge of the screen switched from red to green, marking the start of the execution interval. After the execution interval had expired 1300 ms later, the bar turned red again. An acoustic signal marked the change of colors. During the execution interval, the sequential movement had to be executed within a maximum movement time of 1200 ms. After every execution, participants received feedback about the deviation from the goal values of all three reversals displayed as a bar graph indicating direction and magnitude of errors.

This was followed by 12 familiarization trials during which the feedback was reduced to one predefined reversal (of the three reversals executed). This was done to enable a more unambiguous manipulation of the feedback valence with clear binary categories in the practice phase. The relevant reversal was indicated by a spatial cue (blue rectangle at left, center, or right position on the screen) at the beginning of each trial. The reversal addressed changed every four trials in serial order.

The initial familiarization phase was followed by a pre-test of 12 trials without subsequent feedback presentation and an experimental practice phase with 192 trials (8 blocks of 24 trials) during which EEG was recorded. Based on a performance adaptive bandwidth, qualitative feedback was given for hits (performance within the bandwidth) and for misses (performance outside of the bandwidth). Hits were displayed as green rectangles and the participant earned 200 points, while misses were displayed as red arrows in the direction of the error (if the produced angle was too small, the arrow points downwards, if the angle was too large, the arrow points upwards) without quantitative error information, and the participants lost 100 points. Differences in values for hits and misses are implemented in the experimental design based on the assumption that losses are psychologically weighted twice as much as gains (Tversky & Kahnemann 1991). The target bandwidth for hits was calculated block wise as the median of the absolute error of the previous 12 trials related to the currently addressed reversal, leading to an even distribution of trials over all conditions (cf., Krause et al. 2020). Participants were told that the bandwidth corresponded to the performance of a reference group of a peer sample. The feedback delay was varied block wise, with short delays of 1500 ms (short feedback delay) or long delays of 7000 ms (long feedback delay). The feedback delay corresponds to the interval between the time point of reaching the predefined reversal (e.g., one, two or three) and the time point of augmented feedback onset. With respect to the short feedback delay of 1500 ms, the shortest delay duration that could be implemented in this setup was selected. For a meaningful prediction for error-detection, there must be a condition where the duration of the feedback delay is known. Without this knowledge, the conditions for the short and long feedback delay would differ in terms of temporal predictability as there would be a low predictability in short-delay trials and a high predictability in long-delay trails, at least after 1500 ms until the end of the long delay at 7000 ms. There was a self-determined break after every block. The mean absolute error was presented every 4 trials, and the current score was displayed after each block. The order of blocks with different feedback delays was counterbalanced across participants (cf., Weinberg et al. 2012). The experiment ended with a post-test of 12 trials without subsequent feedback. Participants were informed that monetary rewards of €30, €20, and €10 are given to the top three performances. An example of a practice trial demonstrating the different experimental conditions can be found here: https://osf.io/swj4c).

### Data analysis

 With respect to evaluating the behavioral data, all trials that contained less or more than three reversals were excluded (4.23% of the total number of 4992 trials). Trials in which the movement time of 1200 ms was exceeded, were kept, if they were executed within the execution interval of 1300 ms, for as long as they were structurally correct.

Motor performance in the different feedback delays (1500 ms, 7000 ms) was evaluated based on the movement accuracy and on the movement consistency. To measure movement accuracy, the absolute error in angular degrees was calculated for each trial. Therefore, the absolute differences between the actual and target values were calculated for each movement reversal of the trial. The mean absolute error of the three reversals was used as the absolute error of this trial. The mean absolute error was calculated for each of the 8 practice blocks of 24 trials (4 for the short feedback delay of 1500 ms and 4 for the long feedback delay of 7000 ms). To analyze movement consistency, the variable error was calculated for each of the three reversals as the standard deviation for the 24 trials of each of the 8 practice blocks. Then, the mean variable error of the reversals was calculated.

Analysis of the neural data was done with Brain Vision Analyzer 2.0 (Brain Products, Munich, Germany). Raw EEG data was offline filtered with a 0.3–30 Hz zero phase shift Butterworth filter; line noise was removed using a Notch filter of 50 Hz. The data were re-referenced to an average reference of all active channels. Ocular artifacts were corrected by using the automatic mode of the ocular correction algorithm of the Analyzer based on Independent Component Analysis (ICA). Triggers set by the photodiode were exported and imported again, after feedback valence (positive, negative) and the feedback delay duration (short, long) were defined. The data were segmented time-locked to feedback onset (epochs from − 800 ms to 2000 ms relative to the short and the long feedback trigger) and baseline corrected (-200 ms to 0 ms relative to the trigger). The automatic mode of the artifact rejection algorithm of the Brain Vision Analyzer was used to remove segments containing voltage steps exceeding 200 µV, fluctuations of amplitudes exceeding 250 µV peak to peak, exceeding a maximal amplitude of 200 µV or a minimal amplitude of -200 µV, as well as segments containing activity below 0.5 µV in an interval exceeding 100 ms in an interval of 500 ms before the trigger up to 800 ms after the trigger. Conditions that exhibited a high number of artifacts were manually checked again. Only participants with 70% artifact-free segments per condition were included to maintain a comparable number of trials per participant and condition. For the analysis of the ERPs associated with internal error processing, the data were segmented time-locked to the time point when the relevant reversal was reached (epochs from − 600 ms to 1500 ms relative to the trigger) and baseline corrected (-240 ms to 0 ms relative to the trigger). The settings for the artifact rejection algorithm were the same as for the analysis of the ERPs associated with feedback processing. Overall, 566 segments of a total number of 5,944 were removed. The data was averaged in terms of valence (positive & negative feedback) and of feedback delay (short and long) for each participant. The distribution of the segments across conditions was as follows: 2081 (positive feedback) and 1075 (negative feedback) for the short feedback delay, 2349 (positive feedback) and 1100 (negative feedback) for the long feedback delay. The grand average for each condition was calculated.

In line with previous literature, ERP analysis was done with the FCz electrode measuring the FRN associated with outcome evaluation (Krigolson 2018), and the ERN for error processing (Falkenstein et al. 1991). For Pe amplitudes being maximal at centro-parietal scalp regions, ERP analysis was done with the Cz electrode for this component measuring error awareness (Falkenstein et al. 1991). FRN was analyzed based on mean activity 25 ms around a detected peak in the time window of 200 ms to 300 ms after feedback onset and quantified as the difference between negative and positive feedback (Miltner et al. 1997; San Martin 2012). The ERN was quantified as mean activity 25 ms around a detected peak in a time window of 100 ms up to 200 ms after the relevant reversal was reached. The Pe was analyzed based on mean activity 50 ms around a detected peak in a time window from 200 ms up to 600 ms after the relevant reversal was reached.

Statistical analysis was done with SPSS (IBM Statistical Package for the Social Science), the alpha level was set to 0.05 for all analyses. Additionally, partial eta squared was calculated as effect size for the variance analyses. Follow-up analyses were conducted with paired *t*-tests with Cohen’s *d* as effect size. One-tailed *t*-tests based on directed hypotheses were labeled *t*_1_. All results are given as mean values and standard deviations. *P*-values that are based on multiple comparisons were Bonferroni-Holm corrected. For the behavioral data, two *t*-tests were calculated to compare Pre- and Posttest-performance with the respect to the absolute error and with respect to the variable error. For the ERPs, an ANOVA with repeated measure on *feedback valence* (positive, negative) and *feedback delay* (short, long) was calculated separately for each ERP (FRN, ERN, Pe).

## Results

### Behavioral data

#### Feedback valence

 Participants received positive feedback in 64.52% of the trials in the short feedback delay (*SD* = 12.38; range 38.04–87.37%), and in 64.56% of the trials in the long feedback delay (*SD* = 11.62; range 40.45–84.62%).

#### Movement time

 With an average movement time of 1024 ms (*SD* = 129; range 753–1233 ms), participants were able to execute the motor task within the defined time window of 1200 ms.

#### Performance in the motor task

**Fig. 3:**
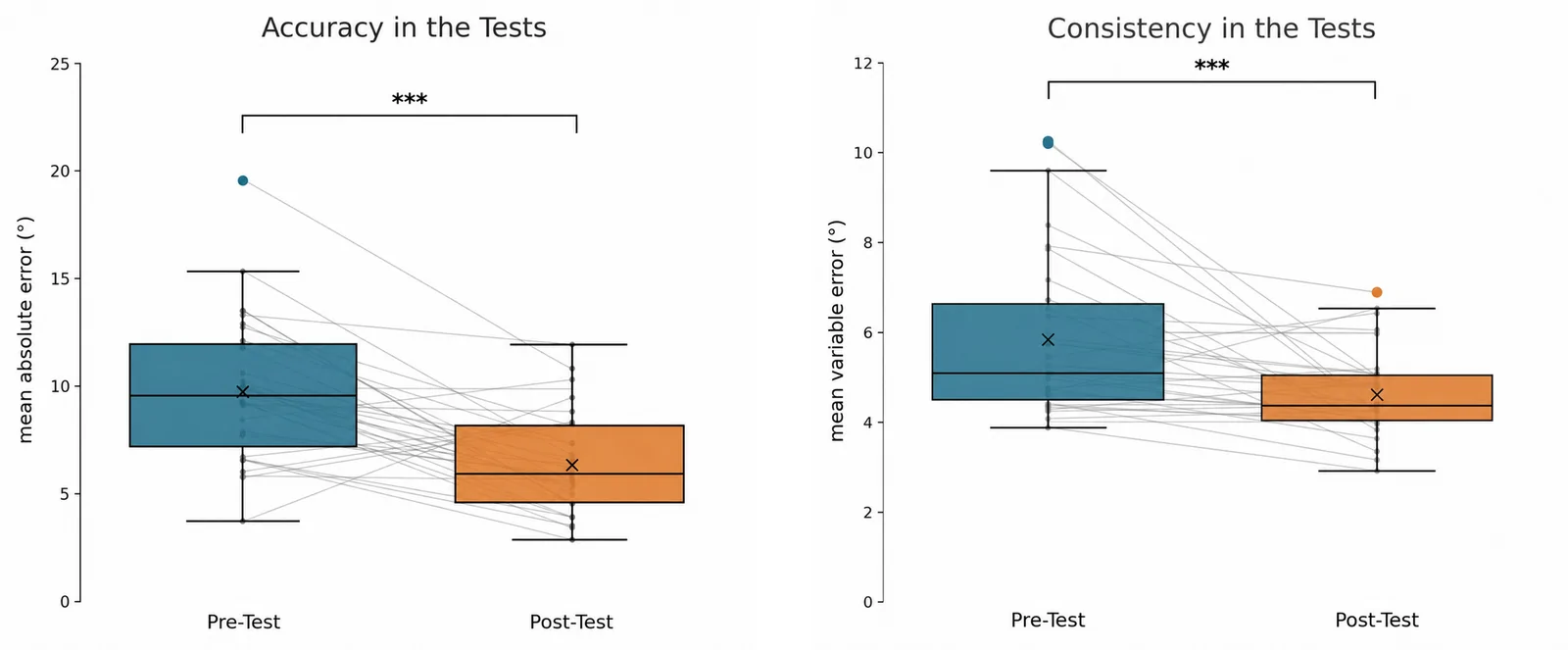
Movement accuracy (absolute error) and movement consistency (variable error) in the tests. The results for motor accuracy (absolute error in angular degrees) are displayed on the left, the results for motor consistency (variable error in angular degrees) are displayed on the right. Individual values of the participants are reflected by the grey lines. The boxes display the median and the 25th and 75th quartiles, whiskers showing the 5th and the 95th percentile. The mean is displayed by a cross and outliers are shown as data points outside of the box for the Pre-test (dark blue) as compared to the Post-test (orange). Significant differences are marked, * *p* < 0.05, ** *p* < 0.01, *** *p* < 0.001.

 For the pre- and posttest without subsequent feedback, the *t*-test for the absolute error revealed a significant difference between movement accuracy in the Pre-test (*M* = 9.80; *SD* = 0.56) as compared to movement accuracy in the Post-test (*M* = 6.48; *SD* = 0.39); *t*_*1*_(1,32) = 5.33; *p* < 0.001; *d* = 0.93 (Fig. 3). *H.AE* can be confirmed. The *t*-test for the variable error also revealed a significant difference between movement consistency in the Pre-test (*M* = 5.84; *SD* = 0.31) as compared to movement consistency in the Post-test (*M* = 4.61; *SD* = 0.16); *t*(1,32) = 3.77; *p* < 0.001; *d* = 0.66 (Fig. 3). Accordingly, *H.VE* can also be confirmed. More information about the motor performance during the practice phase of 192 trials with subsequent feedback presentation can be found in the supplements.

### Neural data

#### FRN

**Fig. 4:**
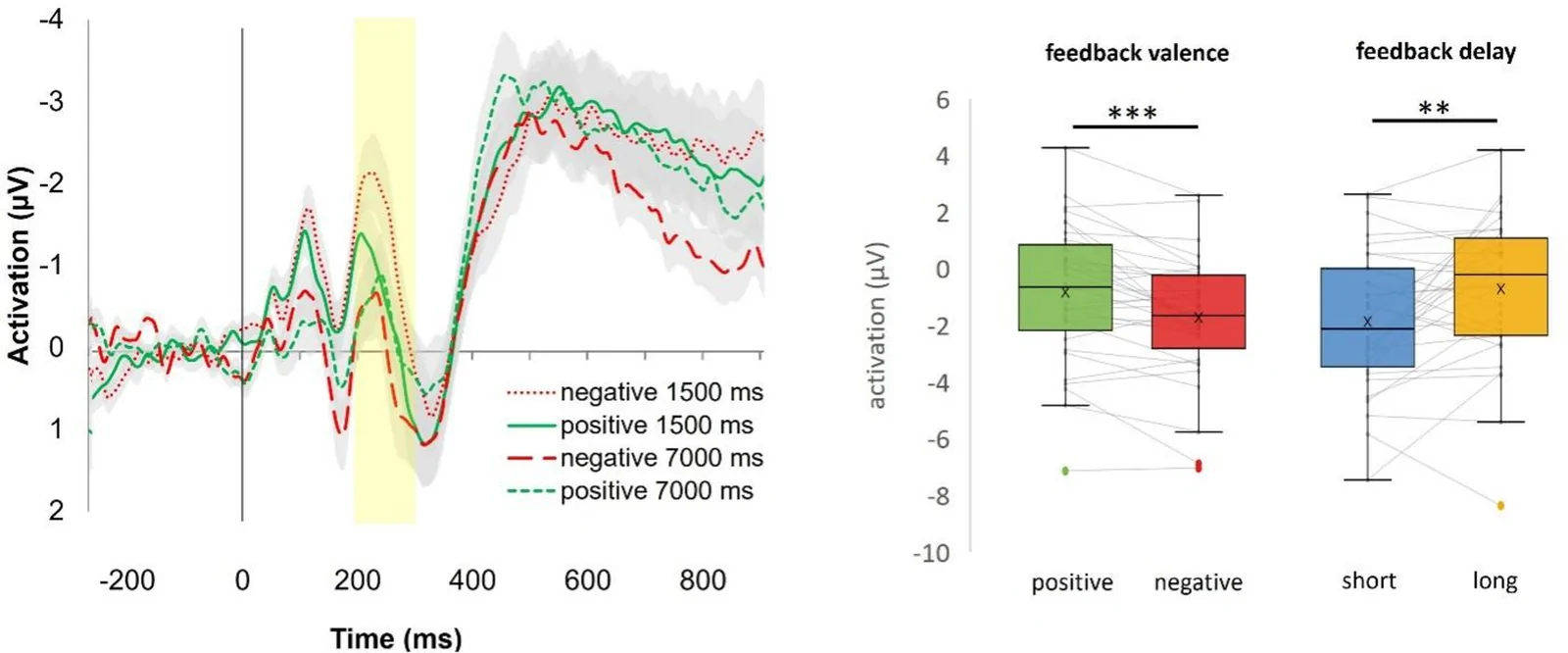
Grand average of the valence-specific waveforms at the FCz electrode and main effects. On the left side, amplitudes of the valence-specific waveforms in microvolts for positive (green) and negative (red) feedback, for the short (solid line and pointed line) and the long (different types of dashed lines) feedback delay, time-locked to feedback onset. The time window of the FRN is highlighted in yellow. On the right side, boxplots depict mean amplitudes in the FRN time window by valence (positive, negative), and by feedback delay (short, long). Individual values of the participants are reflected by the grey lines. The boxes display the median and the 25th and 75th quartiles, whiskers showing the 5th and 95th percentile. The mean is marked by a cross and outliers are shown as data points outside of the box. Significant differences indicated by the ANOVA are marked, * *p* < 0.05, ** *p* < 0.01, *** *p* < 0.001.

 At the FCz electrode, an ERP component with a negative deflection was observed in the FRN time window, with a mean peak latency of 237.39 ms (*SD* = 14.63; range 211 ms – 262 ms) following feedback onset. To verify the presence of an FRN across conditions, a one-sample *t*-test was conducted on the difference wave amplitudes (negative minus positive feedback) collapsed across feedback delays. This analysis showed that the mean FRN amplitude (*M* = -0.90, *SD* = 1.23) differed significantly from zero, *t*(1,32) = -4.18, *p* < 0.001, *d* = -0.73. To examine whether feedback delay duration modulated the FRN, mean amplitudes were analyzed using a 2 (*valence*: positive, negative) x 2 (*feedback delay*: short, long) repeated-measures ANOVA on the valence-specific waveforms (Fig. 4, left side). The ANOVA revealed a significant main effect of *feedback valence*; *F*(1,32) = 17.47; *p* < 0.001; *η*_p_^2^ = 0.35; indicating more negative amplitudes after negative feedback (*M* = -1.62; *SD* = 2.23), as compared to positive feedback (*M* = -0.73; *SD* = 2.44) (Fig. 4, right side). Further, a significant main effect of *feedback delay* was observed; *F*(1,32) = 9.47; *p* = 0.004; *η*_p_^2^ = 0.23; with more negative amplitudes after the short feedback delay duration of 1500 ms (*M* = -1.77; *SD* = 2.45), as compared to the long feedback delay duration of 7000 ms (*M* = -0.59; *SD* = 2.57) (Fig. 4, right side).

**Fig. 5:**
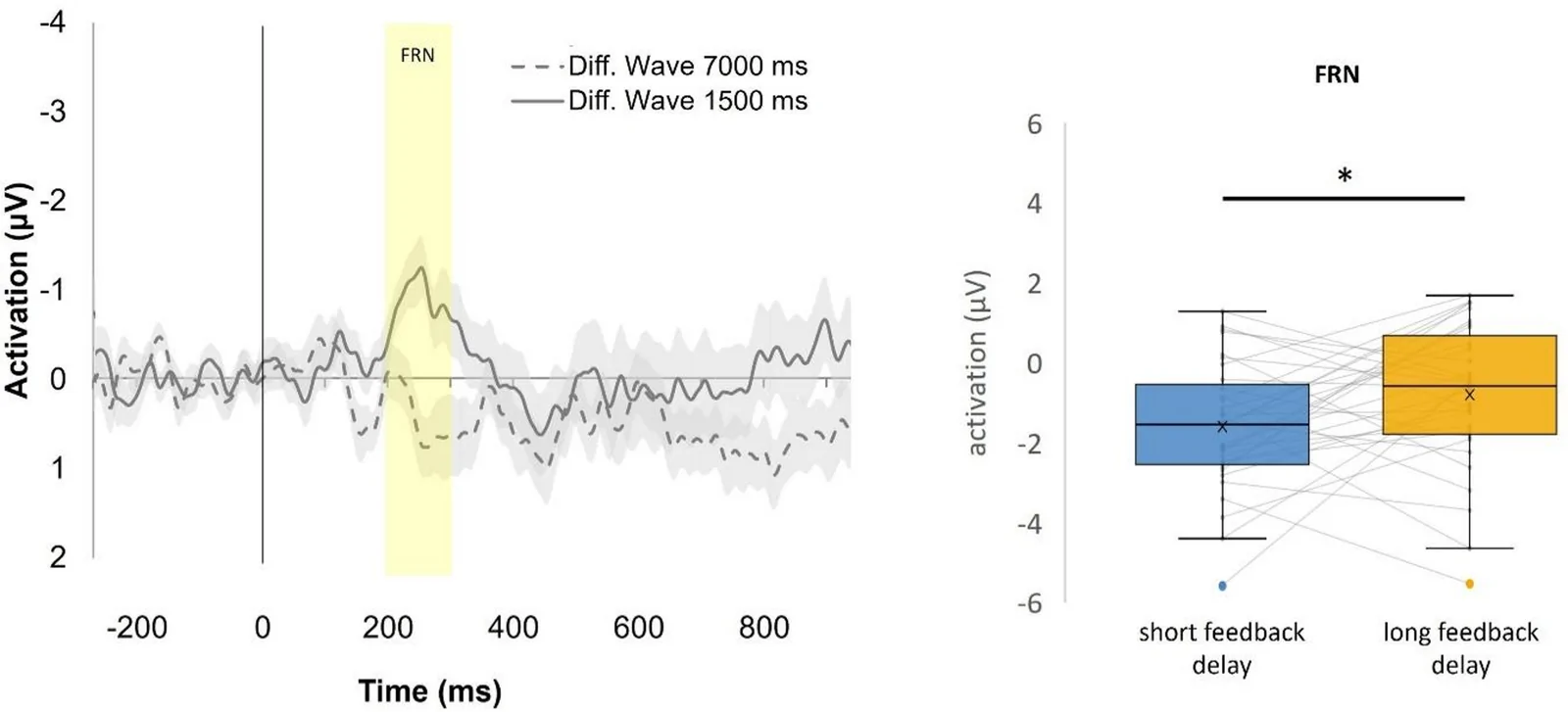
Grand average difference waveforms at the FCz electrode and distribution of the FRN amplitudes. On the left side, the difference waveforms in microvolts which correspond to the FRN (negative minus positive feedback) are shown for the short (solid line) and the long (dashed line) feedback delay, time-locked to feedback onset. The time window (in milliseconds) of the FRN component is highlighted. On the right side, boxplots depict FRN amplitudes for the short (blue) and the long (yellow) feedback delay. Individual values of the participants are reflected by the grey lines. The boxes display the median and the 25th and 75th quartiles, whiskers showing the 5th and 95th percentile. The mean is marked by a cross and outliers are shown as data points outside of the box. Significant differences are marked, * *p* < 0.05, ** *p* < 0.01, *** *p* < 0.001.

However, as the FRN was operationalized as the difference between responses to negative minus positive feedback, the critical hypothesis test was the *valence* x *feedback delay* interaction. This interaction was significant; *F*(1,32) = 4.29; *p* = 0.047; *η*_p_^2^ = 0.12. A post-hoc *t*-test was computed comparing the difference waves of the short and long feedback delay duration (Fig. 5, left side). The analysis revealed that the FRN was more pronounced in the short feedback delay condition (*M* = -1.29; *SD* = 1.54) as compared with the long feedback delay condition (*M* = -0.50; *SD* = 1.74); *t*(1,32) = -2.07, *p* = 0.023, *d* = -0.36 (Fig. 5). Thus, *H.FRN* was confirmed. Feedback delay duration significantly modulates FRN amplitudes.

#### ERN and Pe

**Fig. 6:**
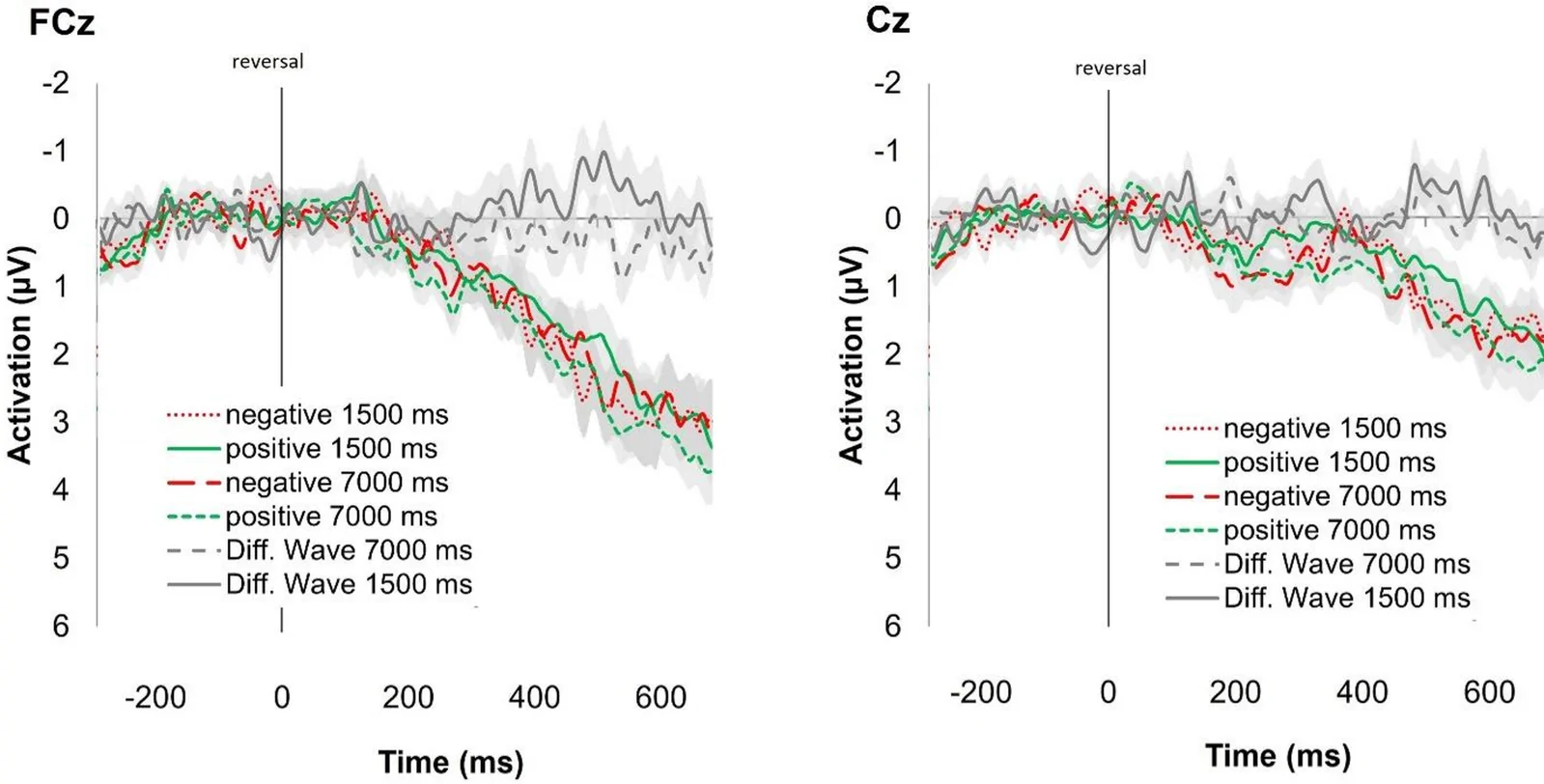
Grand averages at the FCz and the Cz electrode time-locked to reaching of the reversal. Neural activation in microvolts for positive (green) and negative (red) feedback for the short (solid and pointed lines) and the long (different types of dashed lines) feedback delay time-locked to the reaching of the movement point currently addresses. The difference waves are displayed in grey for the short (solid line) and the long (dashed line) feedback delay.

 With respect to the ERN (expected to be measured at the FCz electrode) and the Pe (expected to be measured at the Cz electrode), an ERP component could not be detected (Fig. 6). One-sample t-tests revealed no significant deviation from 0, neither with respect to the ERN (short feedback delay: *t*(1,32) = 0.81; *p* = 0.423; *d* = 0.14; *M* = 0.24; *SD* = 1.70; long feedback delay: *t*(1,32) = 1.75; *p* = 0.090; *d* = 0.30; *M* = 0.73; *SD* = 1.40), nor with respect to the Pe (short feedback delay: *t*(1,32) = 1.24; *p* = 0.225; *d* = 0.22; *M* = 0.35; *SD* = 1.61; long feedback delay: *t*(1,32) = 0.08; *p* = 0.939; *d* = 0.01; *M* = 0.02; *SD* = 1.42). As a consequence, *H.ERN* and *H.Pe* could not be confirmed within the current experimental setting.

## Discussion

The current study investigated neural processing of delayed valence-dependent augmented feedback for two durations (1500 ms, 7000 ms). The emphasis was on the distinct neural correlates that are associated with the processing of outcome feedback (FRN).

## Frequency of feedback valences

The distribution of positive (64.5%) and negative feedback was uneven but comparable across feedback delay conditions. Although a performance-adaptive bandwidth was aimed for approximating 50% per condition, participants received substantially more positive feedback. This should be considered when interpreting the results, as feedback frequency influences both behavior (Krause et al. 2018) and neural processing (Bellebaum et al. 2010; Holroyd & Krigolson 2007). Surprisingly, the adaptive bandwidth did not yield balanced valences as in prior studies (e.g., Krause et al. 2020; Margraf et al. 2022 ), where augmented feedback was more ambiguous, while it was reduced to the reversal with the largest deviation from the goal value without providing the reversals identity. In the current study, feedback addressed a predefined reversal, enabling participants to implement targeted corrections more effectively, thus biasing toward positive feedback, as the bandwidth for positive feedback was set according to the median error value of the preceding practice block.

### Behavioral data

Participants practiced a right elbow extension-flexion sequence with a lever device across 8 blocks of 24 trials (192 total), with qualitative feedback after each trial. Performance changes were assessed in 12 trials without feedback before and after the practice intervention (pre-test – post-test). As hypothesized, movement accuracy (measured by the absolute error) improved significantly from pre-test to post-test (*H.AE* confirmed). The same was true for movement consistency (measured by the variable error), which also improved significantly from pre-test to post-test (*H*.*VE* confirmed). As expected, participants were able to acquire the motor task during a single practice session with augmented feedback in such a way that, even without supplementary feedback information, there was a significant improvement in accuracy and consistency in the Post-test compared to the Pre-test.

### Neural data

With respect to the neural data, the analysis demonstrated a significant effect for the FRN (*d* = -0.73) at the FCz electrode, which however, can be evaluated as medium to strong. In addition, the valence effect in the condition-specific waveforms in the time-window of the FRN was at least lower (*η*_p_^2^ = 0.35) than those documented in the previous studies in a similar setting (Krause et al. 2020: *η*_p_^2^ = 0.42; Margraf et al. 2022 : *η*_p_^2^ = 0.45). The effect sizes of the previous studies were also evaluated as low compared to effect sizes in cognitive studies (e.g., Pfabigan et al., 2014: *η*_p_^2^ = 0.66). The rationale behind this observation was elucidated by the transparency of the bandwidth in the studies mentioned before. In these studies, error information was also contained in the positive feedback display, resulting in feedback conditions that were ambivalent with respect to feedback valence (for a detailed discussion see Margraf et al. 2022). This explanation is not applicable to the present results, as the design of the feedback display was quite different. In the former study, the bandwidth, indicated by a transparent green bar, overlaid the blue error bar. Consequently, besides qualitative information about feedback valence, participants received quantitative information about the direction and the magnitude of the error, as well as about the width of the actual bandwidth. In the present study, augmented feedback provided qualitative information about feedback valence. Quantitative information was reduced to the direction of the error, but only in the negative feedback display. Therefore, clear valence categories can be assumed here.

A first explanation for the smaller effect size for feedback valence, might be an assumably higher predictability of valence. This is because information about the identity of the reversal to which the feedback is assigned is given to the performer, which was not the case in earlier studies in this experimental setting. A second explanation for the smaller effect sizes in the current study could be the unequal distribution of the valence categories. Participants received positive feedback for approx. 65% of the trials and this higher hit frequency could have resulted in smaller FRN amplitudes due to different outcome expectations (Froemer et al. 2016).

However, an examination of the ERP plots revealed that the valence-specific curves already exhibited differences in the preceding P2 component with respect to the longer feedback delay condition (see Fig. 4), which may have modulated the effect in the FRN component (cf. Gheza et al. 2018; Gibbons et al. 2016; Zou et al. 2022). In general, the anterior P2 component is assumed to be larger after stimuli containing target features that are infrequent (Luck 2014). This may be a third reason for the rather small valence effect in the current study. In contrast to the positive feedback display, which only provided information about qualitative feedback valence, the negative feedback display contained also information about the direction of the error. Although this feedback design can be assumed to be practical for motor learning, it creates different levels of complexity with respect to the feedback displays. Therefore, feedback complexity might be a source of confound. Participants might have evaluated negative feedback as more relevant as it provided specific information about how the movement should be adapted. Solving this confound is not trivial, as additional information of error direction in successful trials (error size within the bandwidth) adds a potential source of attributing a negative valence on a subjective level on the one hand, while giving not information on the direction of error has a limited external validity, as this is not representative for motor learning contexts. This aspect is a limitation of the current design and must be kept in mind when discussing the neurophysiological results. Nevertheless, it should be made clear at this point that the results related to feedback valence must be interpreted with caution and are probably not purely valence effects. The results are likely to be overlaid with effects caused by the unequal feedback distribution inducing expectancy effects with higher FRN-amplitudes after less expected feedback categories according to its lower frequency and by the varying informational content of the feedback displays (i.e., valence information in the positive condition and valence information plus direction of error in the negative condition).

With respect to the feedback delay duration, it could be shown that shorter feedback delay durations elicit higher amplitudes of the FRN as compared to longer feedback delay durations. As expected, this suggests that the processing of augmented feedback is less evident in extended feedback delay conditions as compared to shorter ones. This finding can be evaluated as an indication that longer feedback delay durations may be more conducive to the utilization of intrinsic feedback information, while shorter feedback delay durations engage processes associated with reinforcement learning (e.g., Hinneberg and Hegele 2022). This assumption could be further proven by examining neural components associated with the processing of intrinsic feedback information (ERN, Pe).

Longer feedback delay durations are associated with rather explicit processes and as a better precondition for error detection (e.g., Liu and Wrisberg 1997). Therefore, longer feedback delays were expected to induce a higher ERN as well as a higher Pe. In the current experimental setting, neither the ERN, nor the Pe could be detected. The absence of clear ERN and Pe components may stem from several factors: One reason may be the continuous, complex nature of the three-reversal sequential motor task that likely produces overlapping intrinsic error signals, blurring time-locked ERP responses. To further investigate this, the ERPs were analyzed with respect to the 3rd reversal in which the problem of overlapping intrinsic error signals should not be present. The plots can be found in the supplements (Fig. E). This analysis also failed to reveal any clear ERPs. There are no comparative studies that examine intrinsic error processing in a motor sequence task measured by the ERN or the Pe. Despite the existence of studies on sequential tasks, these tend to consist of button presses in the correct order (e.g., Beaulieu et al. 2014). It can be assumed that individual events are more easily distinguished in that kind of task as compared with learning a coordinated motor sequence, as in the current study. However, to verify whether error processing occurs within the anticipated time frame, which may not be reflected in the ERPs, a time-frequency analysis, being less dependent on the temporal distinctness, was also conducted with the expectation that error processing should be reflected in the activity of the frontal theta-band (4–8 Hz) (see supplements). This was not the case.

This does not imply an absence of intrinsic error processing. The lack of explicit error awareness assessment during the experimental session may have contributed, as the Pe amplitude is strongly linked to conscious error recognition (Nieuwenhuis et al. 2001). Further, participant’s attentional focus could have varied, as participants were informed that the feedback had been reduced to one predefined reversal, but still all reversals had to be hit. This instruction was intended to ensure that the movement remained unaltered and was not reduced to a single reversal. The instruction that only the reversal currently addressed accounted for the evaluation of the performance with respect to feedback valence may not have hindered participants from processing information related to the other reversals. Especially, bottom-up processing of motor errors is hard to suppress (Varraine et al. 2002). This might have led to the possibility that some participants focused on the currently addressed reversal, while others put the focus on all three reversals. As a result, the timing of intrinsic error processing was presumably quite different across participants, and the effects may have therefore been averaged out in the neural analyses. Moreover, it seems plausible that there is a tendency to weigh the processing of multiple sources of error information according to their sequential occurrence in a motor sequence. In a follow-up experiment, participants will be required to evaluate their own performance via button press. This might force participants to focus mainly on the reversal that is currently addressed and make intrinsic error processing more consistent across the sample.

Future studies should consider incorporating subjective error awareness reports, tasks that temporally isolate discrete errors to better capture ERN and Pe components or provide explicit instructions to focus on a single error event, which may align intrinsic error processing and improve ERP detectability.

## Conclusion

In general, the behavioral data corresponded to the expected outcomes for a single practice session. Participants demonstrated an improvement in performance, as indicated by enhanced movement accuracy and movement consistency. With respect to the neural results associated with augmented feedback processing, it can be noted that a valence-dependent response could be measured in the expected time-window of the FRN. Further, it should be emphasized that the current results demonstrated the substantial impact of feedback delay on neural processing of augmented feedback. Future research must examine the long-term effects on learning performance with respect to motor automatization.

## Supplementary Information

Below is the link to the electronic supplementary material.

Supplementary Material 1

## Data Availability

All empirical data will be saved in accordance with the open science and open access initiative of the German Society of Psychology (DGPs). Data for this manuscript and supplementary material can be accessed at https://osf.io/swj4c. The assumptions tested in this study were pre-registered prior to data collection (https://aspredicted.org; #225329).
