# Supplementary material for: Effects of augmented feedback delay on neural correlates of feedback processing in motor practice
Source: Exp Brain Res. 2026 Aug 24;244(9):181. doi: 10.1007/s00221-026-07365-z (PMC13503455; doi:10.1007/s00221-026-07365-z)
Supplement: Supplementary file 1 [file 221_2026_7365_MOESM1_ESM.docx]

**Supplements**

**Effects of augmented feedback delay on neural correlates of feedback processing in motor practice**

Linda Margraf^1^, Daniel Krause^1^, Lisa K. Maurer^2^, & Matthias Weigelt^1^

^1^Psychology and Movement Science, Department of Sports and Health, Paderborn University

^2^Training and Movement Science, Physical Education and Sports Science, University of Kassel

Practice Phase Behavioral Results	2

Individual Values of the FRN at the FCz-electrode after Feedbackonset	3

Time-frequency analysis	4

ERP-plots Reduced to the 3^rd^ Reversal	8

**Practice Phase Behavioral Results**

With respect to the absolute error as measurement of movement accuracy during the practice phase, the ANOVA revealed a significant main effect for *block*, *F* = 5.36; *p* < .001; *η*_p_^2^ = .14. The contrasts revealed a linear relationship; *F* = 11.61; *p* = .002; *η*_p_^2^ = .27. Participants improved in movement accuracy from the first block of practice (*M* = 8.39; *SD* = ±3.01) to the last block of practice (*M* = 6.69; *SD* = ±2.69) (Fig. A, on the left side). For movement consistency during the practice phase, the ANOVA also revealed a significant main effect for *block*, *F* = 5.34; *p* < .001; *η*_p_^2^ = .14. Again, a linear relationship can be revealed; *F* = 22.43; *p* < .001; *η*_p_^2^ = .41. Participants improved consistently from the first block of practice (*M* = 6.49; *SD* = ±2.08) to the last block of practice (*M* = 4.96; *SD* = ±1.58) (Fig. A, on the right side).

**Fig. A** Accuracy and consistency during the practice phase. The boxes display the median and the 25^th^ and 75^th^ quartiles, whiskers showing the 5^th^ and the 95^th^ percentile. The mean is displayed by a cross and outliers are shown as data points outside of the box for the mean absolute error in angular degrees for the Pre-test (dark blue) as compared to the Post-test (orange) on the left side, and for the practice blocks (turquoise) on the right side. Significant differences are marked, * p < 0.05, ** p < 0.01, *** p < 0.001.

**Individual Values of the FRN at the FCz-electrode after Feedbackonset**

**Fig. B** Individual values of the FRN. The table on the left shows, in the first column, the values in microvolts following positive feedback in the FRN time window, and in the middle column, the corresponding values for negative feedback. The third column displays the FRN-values sorted by the magnitude of the difference between the two feedback valences. Each row corresponds to one participant. The colors indicate negative and positive polarities of the amplitudes. In the line-plot on the right side the induvial valence-values are displayed, on the left side for positive feedback and on the right side for negative feedback. The thin grey lines connect the two values for one participant, so each line represents one participant. The thick black line displays the group average.

**Time-frequency-analysis**

Due to the relatively small valence-effect in the FRN-component, and a complete absence of the ERN- and the Pe-component, further in-depth analyses were conducted to provide additional insights and explanatory approaches. The processing of error information in a predictive manner (before feedback onset) and in a postdictive manner (after feedback onset) was additionally examined based on the underlying frequency-band activities via time-frequency-analysis with a focus on the activity in the frontal theta-band (4-8 Hz). Research has shown that various frontal ERPs seem to have a share in the frontal theta-band activity, therefore, frontal theta-band activity is interpreted as an indicator that cognitive control is needed to adjust behavior (Cavanagh & Frank, 2014). While Hajihosseini and Holroyd (2013) found correlations between frontal theta power and FRN amplitudes, Luu et al., (2005) reported associations between frontal theta and the ERN. In a recent study, frontal theta-band activity was also examined in a similar setting compared to the current study (Margraf et al., 2023). While the results do not show a correlation with FRN, they do show a correlation with late fronto-central positivity (LFCP), another potential approach to error processing. However, it can be assumed that frontal theta-band activity is a neural signal of error processing, with higher power associated with negative feedback.

Time-frequencies were decomposed using the complex Morlet wavelet transform of the Brain Vision Analyzer. The analysis was based on Gaussian sine waves, a Morlet parameter of 5 in the frequency range from 1 to 20 Hz in 30 logarithmic steps. A baseline normalization (based on a subtraction method) from – 500 to – 100 ms before feedback onset was applied to the data. The total frequency power spectrum was conducted by averaging all segments in terms of valence (positive, negative) and of feedback delay (short, long) for each participant after the Morlet wavelet transform was performed. The phase-locked part of frequency activity (evoked power) was conducted by averaging the segments (as explained before) before the Morlet wavelet transform was performed. The non-phase-locked part of frequency activity was calculated by subtracting the evoked power from the total power (induced power). The time-windows for the analyses were adapted from the time-windows of the respective ERP-components.

**Fig. C** Time-frequency-activities. Total time-frequency activity given in real power values (µV^2^). The feedback valence is marked by rectangles in the upper right corner of each plot, the plots for the short feedback delay can be found on the left side, the plots for the long feedback delay on the right side. Time-frequencies for the FCz electrode time-locked to feedback onset can be found in the upper row, those for the FCz electrode time-locked to the relevant movement reversal in the middle row, and those, for the Cz electrode time-locked to the relevant movement reversal in the bottom row.

Regarding predictive feedback processing, no frequency-specific response could be identified, neither at the FCz electrode (Fig. C, middle row), nor at the Cz electrode (Fig. C, bottom row). With respect to postdictive feedback processing, there was a response in the theta-frequency band about 250 ms after feedback onset (Fig. C, upper row). The 2 (*valence*: positive, negative) x 2 (*feedback* *delay*: short, long) ANOVA of total theta power at the FCz electrode revealed a main effect of *valence*, *F*(1,32) = 15.77; *p* < .001; *η*_p_^2^ = .33. Frontal theta power was higher after negative feedback (*M* = 3.59; *SD* = 2.94) as compared to positive feedback (*M* = 2.21; *SD* = ±2.05). There was no main effect of *feedback delay*, and the interaction between *valence* and *feedback* *delay* was not significant.

For descriptive purposes, the theta-power is plotted separated by evoked and induced power for the FCz electrode (Fig. D). The evoked (phase-locked) theta-power in the middle row corresponds to the ERP, while the induced (non-phase-locked) theta-power displays the part of the theta power that could not be captured by the ERP.

**Fig. D** Time-Frequency plots for the FCz electrode, displaying the frequency range from 1 Hz to 20 Hz within a time window from -500 to 1500 ms around feedback onset (0 ms) given in real power values (μV^2^). Feedback conditions (positive, negative) are marked by colored rectangles (green, red) on the upper right corner of each plot. Plots for the short feedback delay are displayed on the left, plots for the long feedback delay are placed on the right. The total frequency power spectrum can be found in the top row, the phase-locked frequency spectrum (evoked power) can be found in the middle row, and the non-phase-locked frequency spectrum (induced power) can be found in bottom row.

References

Cavanagh, J. F., & Frank, M. J. (2014). Frontal theta as a mechanism for cognitive control. *Trends in Cognitive Sciences*, *18*, 414–421. https://doi.org/10.1016/j.tics.2014.04.012

Hajihosseini, A., & Holroyd, C. B. (2013). Frontal midline theta and N 200 amplitude reflect complementary information about expectancy and outcome evaluation. *Psychophysiology*, *50*, 550–562. <https://doi.org/10.1111/psyp.12040>

Luu, P., Tucker, D. M., & Makeig, S. (2004). Frontal midline theta and the error-related negativity: neurophysiological mechanisms of action regulation. *Clinical Neurophysiology*, *115*(8), 1821-1835. <https://doi.org/10.1016/j.clinph.2004.03.031>

Margraf, L., Krause, D., & Weigelt, M. (2023). Frontal theta reveals further information about neural valence‐dependent processing of augmented feedback in extensive motor practice—A secondary analysis. *European Journal of Neuroscience*, *57*(8), 1297-1316. <https://doi.org/10.1111/ejn.15951>

**ERP-plots Reduced to the 3^rd^ Reversal**

Unfortunately, the ERN (measured at the FCz) and the Pe (measured at the Cz) as indicators of intrinsic error processing seemed not to be detectable in the current setting. One explanation might be based on the structure of the task which held the risk of an overlap of successive components. The motor task consisted of 3 movement reversals (70°, 20°, 70°), which should be hit within a maximum movement time of 1200 ms. So, participants had about 400 ms to reach a reversal, this was also the approximate time between two successive movement reversals (reversal one to two, and reversal two to three). Although the participants received augmented feedback for only one predefined reversal, each of the three reversals could provoke ERPs of intrinsic error processing (ERN, Pe). To prove this, the neural activity was additionally plotted only for the last reversal (third reversal) for which the assumption of overlapping components should not be true (Fig. E).

Cz

3rd reversal

FCz

3rd reversal

**Fig. E** Grand averages reduced to the third reversal. The vertical line at 0 ms reflects the point, the reversal was hit. On the left side, amplitudes in microvolts at the FCz-electrode were displayed. Here, the ERN should be detected. On the right side, amplitudes in microvolts at the Cz-electrode were shown. The Pe should be detected at this electrode. The condition specific waveforms are marked by the colors: negative feedback in the long feedback delay condition = black, negative feedback in the short feedback delay condition = red, positive feedback in the long feedback delay condition = blue, positive feedback in the short feedback delay condition = green.
